# Feasibility Study Comparing Physical Activity Classifications from Accelerometers with Wearable Camera Data

**DOI:** 10.3390/ijerph17249323

**Published:** 2020-12-13

**Authors:** Alyse Davies, Margaret Allman-Farinelli, Katherine Owen, Louise Signal, Cameron Hosking, Leanne Wang, Adrian Bauman

**Affiliations:** 1 Nutrition and Dietetics Group, Charles Perkins Centre, School of Life and Environmental Sciences, The University of Sydney, Sydney, NSW 2006, Australia; margaret.allman-farinelli@sydney.edu.au (M.A.-F.); lwan4745@uni.sydney.edu.au (L.W.); 2 Prevention Research Centre, School of Public Health, The University of Sydney, Sydney, NSW 2006, Australia; katherine.owen@sydney.edu.au (K.O.); adrian.bauman@sydney.edu.au (A.B.); 3 Health Promotion & Policy Research Unit, Department of Public Health, University of Otago, P.O. Box 7343, Wellington South, Wellington 6242, New Zealand; louise.signal@otago.ac.nz; 4 Transformational Bioinformatics Group, Commonwealth Scientific and Industrial Research Organization, North Ryde, Sydney, NSW 2113, Australia; Cameron.Hosking@csiro.au

**Keywords:** methods, physical activity, compendium, wearable cameras, accelerometer, activity intensities, sedentary behavior, measurement, young adults, public health

## Abstract

Device-based assessments are frequently used to measure physical activity (PA) but contextual measures are often lacking. There is a need for new methods, and one under-explored option is the use of wearable cameras. This study tested the use of wearable cameras in PA measurement by comparing intensity classifications from accelerometers with wearable camera data. Seventy-eight 18–30-year-olds wore an Actigraph GT9X link accelerometer and Autographer wearable camera for three consecutive days. An image coding schedule was designed to assess activity categories and activity sub-categories defined by the 2011 Compendium of Physical Activities (Compendium). Accelerometer hourly detailed files processed using the Montoye (2020) cut-points were linked to camera data using date and time stamps. Agreement was examined using equivalence testing, intraclass correlation coefficient (ICC) and Spearman’s correlation coefficient (rho). Fifty-three participants contributing 636 person-hours were included. Reliability was moderate to good for sedentary behavior (rho = 0.77), light intensity activities (rho = 0.59) and moderate-to-vigorous physical activity (MVPA) (rho = 0.51). The estimates of sedentary behavior, light activity and MVPA from the two methods were similar, but not equivalent. Wearable cameras are a potential complementary tool for PA measurement, but practical challenges and limitations exist. While wearable cameras may not be feasible for use in large scale studies, they may be feasible in small scale studies where context is important.

## 1. Introduction

The benefits of being physically active and reducing sedentary behavior are well documented [1]. Rates of chronic disease are increasing, and many young adults are gaining weight faster than ever before [2], suggesting that daily physical activity (PA) levels have become an important public health priority. For general health and quality of life, the World Health Organization (WHO), recommends at least 150 min of moderate intensity activity throughout the week or at least 75 min of vigorous intensity activity [3]. Moderate intensity is defined as any energy expenditure (EE) of at least 3.0 times the resting level or 3.0 metabolic equivalents (METs) whereas vigorous intensity is at least 6.0 times the resting level or 6.0 METs [3]. The upper limit for additional health benefits is the accumulation of 300 min of moderate intensity aerobic PA or 150 min of vigorous intensity aerobic PA per week [3]. Valid and reliable assessment of habitual PA in free-living settings is important for epidemiological research as well as understanding contextual aspects of PA patterns to inform policy and practice [4].

Device-based assessments are frequently used to measure PA in a range of settings including free-living settings [5,6]. While accelerometers lack contextual information, they provide information on EE as well as information about the frequency, duration and intensity of PA [7]. To translate accelerometer output data, cut-points are applied to the activity counts per defined period of time (i.e., an epoch) to convert activity counts to activity intensity categories of sedentary, light or moderate-to-vigorous physical activity (MVPA) [8]. The total minutes spent at each activity level is highly dependent on which cut-point is selected and several have been proposed for adults [9,10,11]. With the increase in use of wrist-worn accelerometers, vector magnitude cut-points for classification of activity intensity from a wrist-worn accelerometer in free-living adults have recently been proposed [12].

While advanced technologies in the field of computer science have shown accurate activity recognition results using wearable sensors (e.g., accelerometers, gyroscopes and magnetometers) [13], current accelerometer models used in PA research and surveillance fail to collect domain-specific PA levels (work, transport, domestic and leisure-time) which is important to inform future public health interventions and policy. Furthermore, they may not provide valid data on some common activities (e.g., cycling, muscle strengthening exercise, static exercise or carrying heavy loads) [14]. For these reasons, new device-based methods may be useful, and one infrequently explored option is the use of wearable cameras. Wearable cameras provide an objective measurement with an advantage that the data captures detailed contextual information on PA carried out in free-living settings. In this study, we are comparing wearable camera data to the accepted objective method of assessing PA, accelerometer data. We are assessing the camera as the test measurement, against an established method (accelerometer), which defines the latter as a putative “reference measurement”. We are not implying that accelerometers are a true “reference measurement”, as there is no easily usable criterion measure for assessing EE, except doubly labelled water, which is less feasible in field studies.

The wearable camera takes continuous digital images from a first-person perspective [15] and based on the image data, assumptions can be made about the activity category, and a MET value can be assigned based on the activity sub-category defined by the 2011 Compendium of Physical Activities (Compendium) [16]. As every image is time and date stamped, it can be matched to accelerometer data to investigate the agreement of this novel method. Wearable cameras have been previously used for travel research [17,18,19], categorization of type and context of accelerometer identified episodes of PA [20] and improving classifications of sedentary behavior [21]. Wearable cameras have also demonstrated feasibility in a pilot study using 24 h time-use diaries [22] and were used as a reference measurement in a validation study [23]. Feasibility is a common term and a common way of describing preliminary studies where the test measurement (wearable cameras) have not been assessed, and this study is assessing the feasibility, utility and challenges in wearable camera use in PA measurement. This is a feasibility study as it makes no assumptions that the test measurement can be used in large scale research but testing this in an initial study is good practice prior to recommending any new measurement assessment technique. The current study compares intensity classifications from accelerometer-based cut-points for classifying sedentary, light and MVPA with wearable camera images in young adults.

## 2. Materials and Methods

### 2.1. Sample

Recruitment methods in addition to inclusion and exclusion criteria were outlined in the larger study protocol [24]. The sub-sample including demographic questions have been described in a previous study [25] and self-reported weight and height measurements have been validated [26]. After all study requirements were completed, participants received a 110 Australian Dollar (AUD) gift voucher as a reimbursement for their time. Ethics approval was obtained by the Sydney University Human Research Ethics Committee (project 2016/546).

### 2.2. Accelerometer

Additional information was required for accelerometer initialization which included: date of birth (day/month/year); non-dominant hand (right, left); ethnicity (White/Caucasian, Black/African American, Asian/Pacific Islander, Latino/Hispanic, other). Participants wore an Actigraph GT9X link wrist-worn accelerometer on their non-dominant hand that was time synchronized with the Autographer camera for three consecutive days. The sample rate was set at 30 Hz. The Actigraph GT9X Link features a programmable liquid-crystal display (LCD) window that can be configured to display the date and time and/or provide real-time activity feedback. We set the display to show 24 h time only. Actilife software (version 6.13.4, ActiGraph, Pensacola, FL, USA) was used to initialize and download accelerometer data. AGD files were saved in both 60- and 30-s epoch length.

### 2.3. Autographer Camera

The Autographer camera captures images from the wearer’s point of view at approximately 30-s intervals on the medium capture rate when switched on (two images per minute). The camera is “matchbox sized” and weighs 58 g. The camera is worn on a lanyard around the neck, adjusted to chest level, for as long as possible during all waking hours. Instructions were given to charge the camera overnight and to carry the portable charger throughout the day as a back-up, given the average battery life of 14 h. Participants were advised to go about their everyday free-living activities. The privacy lens allowed participants to halt the recordings temporarily (i.e., bathroom) or if individuals felt uncomfortable having their image captured. The camera was removed during bathing and swimming activities. We asked participants to leave the camera on during MVPA but if contact with other people was likely (i.e., football), participants could remove the camera. Following data collection, participants were provided the opportunity to review and delete images before submission to the researchers.

### 2.4. Reliability Testing

Inter-rater agreement testing was conducted (AD & LW) on a test dataset of 322 images for the following dimensions: PA domain (100%), posture (100%) and activity intensity (96%). A 90% agreement threshold presumes acceptable inter-rater agreement [27].

### 2.5. Image Coding

**Figure 1:**
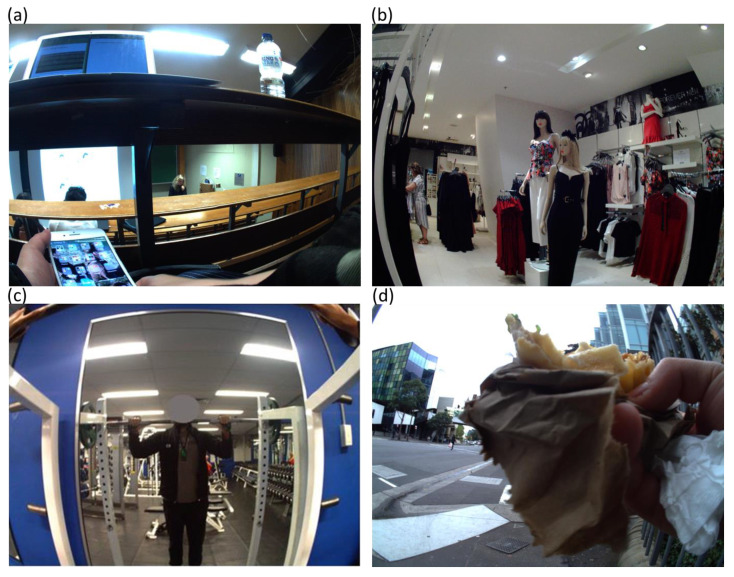
Sample coding using wearable camera images of sedentary, light and moderate-to-vigorous physical activity (MVPA). (**a**) image code: domain, occupational; posture, sitting/reclining; activity category, miscellaneous; activity sub-category, 09055 sitting, talking in person, on the phone, computer, or text messaging, light effort, metabolic equivalent (MET) 1.5. (**b**) image code: domain, leisure; posture, standing; activity category, home activity; activity sub-category, 05065 non-food shopping, with or without a cart, standing or walking, MET 2.3. (**c**) image code: domain, leisure; posture, standing; activity category, conditioning exercise; activity sub-category; 02054 resistance (weight) training, multiple exercises, 8–15 repetitions at varied resistance, MET 3.5. (**d**) image domain, transport; posture, standing, activity category (1), walking; activity sub-category (1), 17270 walking, to work or class, MET 4.0; activity category (2), self-care; activity sub-category (2), 13035 talking and eating or eating only standing MET 2.0.

An image coding schedule was developed and refined accordingly until all members of the research team agreed. The full coding manual is available from the first author on request. A custom application was designed for the purpose of coding PA in four dimensions: (1) PA domain (occupational, domestic, transport and leisure); (2) posture (lying, sitting/reclining, standing, changing position); (3) activity category (21 activities); and (4) activity sub-category (821 activities) with the associated MET value defined by the Compendium [16]. The application was piloted on camera images from two participants. Image coding (including preliminary coding) took place between June 2018 and January 2020 and detailed annotation was applied to 78 participant records by one researcher (AD). Sedentary behaviors were considered those requiring ≤1.5 METs Figure 1a. Light activity were images between 1.6–2.9 METs Figure 1b. Due to the difficulty in differentiating between images that were moderate or vigorous, MVPA were combined and considered those requiring ≥3.0 METs Figure 1c. If two or more activities were coded in a given image, the application csv output showed the highest MET value Figure 1d. In cases where the activity was not in the Compendium, a similar activity was chosen. For example, drinking was matched with the appropriate eating code depending on whether the subject was sitting or standing. If the image could not be coded (e.g., blurry), the previous or subsequent image was compared and if deemed similar, the image code was copied, otherwise no code was applied.

### 2.6. Data Analysis

The 30 Hz data were transformed to counts using the normal filter in Actilife. Accelerometer data were collected using 30-s epochs and then scaled to 60-s epochs before applying the cut-points. The 30-s epoch length was chosen so it could be matched to the camera images which were taken every 30 s. Accelerometer non-wear was defined by an interval of at least 60 consecutive minutes of zero activity intensity counts and was determined using the Actilife Troiano default setting [28]. Hourly detailed files were downloaded and processed using Actilife, applying cut-points for Montoye (2020), suggested for wrist worn accelerometry [12]. The cut-points were counts/min (CPM): <2860 (sedentary), 2860–3940 (light) and ≥ 3941 (MVPA). The hourly summary data were then linked to the camera data using date and time stamps. The time spent (min/h) as sedentary, light or MVPA coded by the camera was compared to the classification from the accelerometer. Daily camera wear was calculated as a total number of images captured per person divided by 120 (as this is the approximate number of images captured per hour as two images were taken per minute) and divided by three (three study days). When more than two images were captured per minute, only the first two images were included. For inclusion in the final analysis, wear-time for both accelerometer and camera needed to be ≥50 min/h. The proportion of time for both devices was calculated as hourly minutes per activity intensity divided by hourly wear-time.

### 2.7. Statistics

Means (95% CI) and equivalence testing was conducted using two one sided *t* tests to determine if mean differences were within a predefined interval [29]. The two assessments were considered equivalent if the camera mean minutes and respective 95% CI were within a 10% interval of the accelerometer mean minutes. The intraclass correlation coefficient (ICC; two way mixed; consistency) was used for reliability between methods (min/h) for each activity intensity. Correlation between the two methods was measured using Spearman’s correlation coefficients (rho). Median and interquartile range (IQR) were used to report device wear-time. Statistical analyses were conducted using SPSS software, version 24.0 for windows (IBM, Armonk, NY, USA). The significance level was set at 0.05.

## 3. Results

A total of 281,041 images were coded and 230,166 included in the final analysis. Of the excluded images, 9474 (4%) were uncodable and 41,401 (18%) exceeded the first two images of each minute. In total 5402 person-hours were linked using 78 participant records. The minimum wear-time for both the accelerometer and camera was set to 50 min/h, resulting in a sample size of 53 participants contributing 636 person-hours to the final analysis with some participants contributing more hours of data. Median (IQR) wear-time/hour for this sample was 60 (60–60) min for the accelerometer and 58 (55–59) min for the camera.

Sample characteristics and hour data contribution is presented in Table 1. The sample included 49% males with a mean (SD) age 24 (4) years and BMI 25.8 (5.4) kg/m^2^. Participants classified as overweight or obese comprised 47% of the sample, comparable to the 46% reported in the 2017–2018 National Health Survey [30]. Two thirds of the sample were from higher socioeconomic, metropolitan areas and 72% had a tertiary education qualification, higher than the 56% reported by the Australian Bureau of Statistics Census [31].

Table 2 shows the Mean (95% CI), equivalence testing, ICC and Spearman’s rho for sedentary, light and MVPA. The mean sedentary behavior estimated by the accelerometer was 42 min/h and resulted in an associated region of equivalence of 37 min/h and 46 min/h for the lower and upper limits, respectively. The estimates of sedentary behavior from the two methods were similar as the 95% CIs for sedentary behavior estimated by the camera fell within this region (M = 34, 95% CI 29, 39), but not equivalent (*t* = −22.3; *p* = 1.00). A good degree of reliability was found between the camera and accelerometer for sedentary behavior, Spearman’s rho = 0.77; average ICC 0.81 (95% CI 0.78,0.84). The mean light intensity estimated by the accelerometer was 12 min/h and resulted in an associated region of equivalence of 11 min/h and 13 min/h for the lower and upper limits, respectively. The estimates of light activity from the two methods were similar as the 95% CIs for light activity estimated by the camera fell within this region (M = 18, 95% CI 13, 23), but not equivalent (*t* = 11.8; *p* = 1.00). Moderate reliability was found between the camera and accelerometer for light intensity activity, Spearman’s rho = 0.59; average ICC 0.55 (95% CI 0.48,0.62). The mean MVPA estimated by the accelerometer was 7 min/h and resulted in an associated region of equivalence of 6 min/h and 7 min/h for the lower and upper limits, respectively. The estimates of MVPA from the two methods were similar as the 95% CIs for MVPA estimated by the camera fell within this region (M = 5, 95% CI 2, 8), but not equivalent (*t* = −5.8; *p* = 1.00). A moderate degree of reliability was found between the camera and accelerometer for MVPA, Spearman’s rho = 0.51; average ICC 0.52 (95% CI 0.44,0.59). The agreement between camera and accelerometer data was also assessed using the traditional Freedson (1998) accelerometer cut-points and these are shown in Supplementary Table S1. When we used the traditional Freedson (1988) cut-points for accelerometry CPM; 0–99 (sedentary); 100–1951 (light); ≥1952 (MVPA) [11], we found a moderate agreement for sedentary behavior (rho = 0.66) and MVPA (rho = 0.55), but low reliability for light intensity activity (rho = 0.27).

Table 3 shows the total minutes of sedentary, light and MVPA for the camera and the accelerometer with the proportion of time spent in each activity intensity. A higher proportion of time (%) was coded as sedentary using the camera images and accelerometer data, respectively, (59,69) compared to light (32,20) and MVPA (9,11).

## 4. Discussion

This study tested the feasibility of using wearable cameras in PA measurement by comparing intensity classifications from accelerometers with wearable camera data in young adults. A moderate to good degree of reliability was found between the camera and accelerometer for sedentary behavior, light intensity activity and MVPA. The estimates of sedentary behavior, light activity and MVPA from the two methods were similar, but not equivalent. Wearable cameras were shown to be feasible for use in small scale studies where context is important. The advantage over accelerometer data is that contextual information such as domain-specific PA levels and type-specific activities can be identified. Such contextual information may help to identify errors in PA intensity classification which may arise from the use of different threshold cut-points and may help to characterize usual behavior by context and setting.

Sedentary behavior is a population wide health priority as it contributes to the risk of ill health and mortality [34]. While good reliability was found between the camera and accelerometer for sedentary behavior (rho = 0.77), the accelerometer recorded higher mean sedentary min/h. The mean proportion of time coded as sedentary (59%) and light activity (32%) by the camera differed from accelerometer classifications of sedentary (69%) and light activity (20%). Despite the wrist-worn accelerometers having higher compliance, there is limited guidance or consensus for cut-points, with further research required to improve the accuracy of PA assessment from wrist-worn devices. The misclassification could be due to the sedentary cut-point threshold used for sedentary behavior (<2860 CPM), but it is important to note that these are the only user friendly cut-points proposed for wrist-worn accelerometers in free-living adults [12]. Other proposed cut-points for MVPA [35] and sedentary behavior [36] lack user friendly methods using raw vector data from the wrist. A previous study using a hip-worn accelerometer was able to correctly classify sedentary behavior 90% of the time using the 100 CPM cut-point with camera images [21]. Alternatively, light intensity activity while sitting may not be captured by the static camera images (i.e., fidgeting). Fidgeting may reduce the health risk during long periods of sitting, independent of PA levels [37]. While the PA Compendium code for fidgeting general or fidgeting hands is categorized as sedentary, “fidgeting feet” is considered light intensity activity [16]. As the camera is positioned at chest level, the lower limb was often missed by the camera images particularly while sitting.

A moderate degree of reliability was found between the camera and accelerometer for light intensity activity (rho = 0.59). Wearable cameras are particularly useful in providing contextual data to help categorize light intensity activity. As accelerometers do not provide information on body position such as the difference between sitting and standing still, previous studies showed that sedentary and light activity intensities may be misclassified using the 100 CPM cut off [21,38]. While contextual information in images may be helpful in corroborating or correcting accelerometer data, visual interpretation by researchers may be limited when faced with estimating speed of movement (i.e., walking). Walking can be classified as either light activity or MVPA and consequently, it is possible that images of participants walking may be misclassified, potentially contributing to the discrepancy in our results. Distance in images has been studied showing promising results by creating depth estimation of single images [39]. This highlights the benefits of using artificial intelligence to estimate speed of movement from images. Substantial challenges exist to correctly classify light intensity activity using the accelerometer cut-point approach. A range of different cut-points are used to categorize accelerometer data and depending on which cut-point is selected, the estimated time spent in different PA intensities differs [40]. Accelerometers are often used as a reference measurement in validation studies for PA research but previous research has highlighted the limitations [14] or suggested that accelerometers may not be the most appropriate reference measurement in free-living settings [41].

Wearable cameras can identify type-specific activities and may provide slightly more reliable and valid estimates for activities such as cycling, resistance and static exercise. These common activities have been shown to underestimate activity counts with the wrist-worn and hip-worn accelerometer [14]. Results from this study comparing camera and accelerometer data reported a moderate degree of reliability for MVPA (rho = 0.51), similar to MVPA correlations with time-use diaries and accelerometers (rho = 0.45–0.69) [41]. Furthermore, a recent validation study reported similarity between activities reported in time-use diaries and camera image data with a strong correlation for the category PA of 0.79 [23]. The accelerometer in our study however, recorded higher mean min/h and mean proportion of time compared to the camera. An explanation for lower MVPA minutes for the camera compared to the accelerometer could be either misclassification of light or MVPA (i.e., walking) or the removal of the camera during MVPA due to possible interference or unwieldiness. A previous pilot study reported minimal occasions of device removal or deactivation but did report that the most frequent issue with wearing the camera during PA was excessive movement (i.e., swing on lanyard) [22].

Despite the PA Compendium being a useful option for estimating METs of activities, it presented some challenges for wearable camera research [16]. Firstly, differences in PA intensities were observed between activity categories for a similar activity. For example, for the activity category occupation, the activity sub-category 11600, standing tasks, light effort (e.g., standing and talking at work) has been assigned MET 3.0; however, for the activity category miscellaneous, the activity sub-category 09055, standing, talking in person, light effort has been assigned a MET 1.8. By selecting the activity sub-category 11600, an MVPA intensity will be applied which may overestimate MVPA unless the individual uses their upper body such as hand gestures during a conversation. Secondly, some light intensity activities in the Compendium may be sedentary depending on the degree of movement. For example, for Figure 1a, the code applied to this image was: domain, occupational; posture, sitting; activity category, miscellaneous; activity sub-category, 09055, sitting, talking in person, on the phone, computer, or text messaging, light effort, MET 1.5. If the participant was not on the phone, this image could have been coded as either sub-category 09065, sitting in class, general, including note-taking or class discussion MET 1.8 or 09040, sitting, writing, desk work, typing MET 1.3. This highlights the need for sedentary codes to be developed in the Compendium for sitting in class, lecture or studying. Thirdly, certain activities identified from the images did not have an associated Compendium code (i.e., karaoke, scooter not motorized and drinking); therefore, were matched to similar activity codes which were discussed and resolved by the research team. Finally, wearable camera annotation from a previous study suggested different activity intensity categories for certain Compendium codes [42]. For example, the Compendium code for the activity category transportation, activity sub-category, 16010 automobile or light truck (not a semi) driving MET 2.5 should be considered for re-classification as sedentary. For this study, the decision was made to adhere to the published Compendium MET values however, the proportion of images coded in this study as driving was small (4% of the total included images).

A strength of this study is that wearable cameras allowed for direct observation of the behaviors examined from a first-person perspective. Two objective methods were compared, limiting recall bias. Inter-rater agreement was high, similar to a previous study [43]. Detailed annotation in four dimensions including the PA domain, posture, activity categories and activity sub-categories revealed potential misclassifications for wrist-worn accelerometers using count-based PA measures to classify PA intensity. While traditional observational methods are intensive during data collection, this is automated when using wearable cameras, but more work is required when coding. As hundreds of thousands of images were manually coded for this study, it may be possible to train and validate machine learning algorithms to reduce the researcher burden for future studies using wearable camera data [44]. Furthermore, with ongoing development in advanced technologies in the field of computer science (i.e., machine learning and deep learning), future studies measuring PA have the potential to adopt such technologies which will further the measurement field [13]. Finally, using cut-points for classification of activity intensity from a non-dominant hand wrist-worn accelerometer in a free-living setting were used which may help to improve the accuracy of PA assessment for wrist-worn devices.

Wearable cameras have some limitations. Firstly, there are feasibility issues with wide-scale adoption of the current wearable camera. Adherence to camera protocols can be improved, as 25 participants were excluded from the analysis due to usable data being less than 50 min/h over the three study days. Further, some participants contributed more data than others depending on hourly device wear-time; however, the data hour contribution was evenly spread for most sample characteristics. Setting the camera at intervals of 5 s or less [43] compared to the 30 s intervals used in this study would allow the coders to better estimate activity intensity, particularly when walking unless depth estimation was applied to single images to estimate speed. It is possible that wear-time could be improved if the size of the camera could be reduced without decreasing battery life. The use of eButton cameras or the use of video [45] are suggested for future studies involving PA. Finally, the wear-time in hours was not exactly the same among participants, and participants contributed to total person-time, but this feasibility study was not estimating proportions meeting PA guidelines, but simply examining fine-grained behavior in specific hours between the two devices.

## 5. Conclusions

This study tested the feasibility of using wearable cameras in PA measurement by comparing accelerometer-based cut-points for classifying sedentary, light and MVPA with wearable camera images. This work shows that wearable cameras are a potential complementary tool for PA measurement but, like all methods, practical challenges and feasibility limitations exist. It is unlikely that wearable cameras will become a surveillance measurement tool in the near future for use in large scale studies; testing them informs a better understanding of their feasibility as technology and artificial intelligence systems improve. This study has shown that wearable cameras may be feasible in small scale interventions or prevalence studies where assessing context is important. An advantage of wearable cameras over other measurement methods such as accelerometry is that simultaneous domain and contextual information can be collected. Such contextual information may help to identify errors in PA intensity classification and better characterize patterns of PA behaviors in context. Reliability was moderate to good between the two objective methods and equivalence was similar, suggesting that the recently proposed vector magnitude cut-points for wrist-worn accelerometers are acceptable for use in a free-living setting. An updated review of the PA Compendium is suggested to include additional activities as well as correct the differences for MET values of similar activities.

## Figures and Tables

**Table 1:** Sample characteristics and data contribution.

Sample Characteristics	Participants (*n*)	Data Contribution (Hours)
**Sex**		
Male	26	335
Female	27	301
**Age (years)**		
18–24	27	318
25–30	26	318
**Body Mass Index (kg/m^2^)**		
Under/Normal < 24.99	28	316
Overweight/Obese ≥ 25.00	25	320
**Socio-economic status ^1^**		
High	33	327
Low	20	309
**Highest education attainment**		
Secondary school or less ^2^	15	180
Trade/Diploma/Apprenticeship	10	131
University degree	28	325
**Geographic location (ARIA) ^3^**		
Metropolitan	32	352
Non-metropolitan	21	284
**Ethnicity**		
White/Caucasian	35	426
Asian/Pacific Islander	12	160
Other	6	50

^1^ Socio–economic status assessed using residential postcode to assign the index of relative socio–economic advantage and disadvantage centile employed within Australia, lowest five deciles = lower, highest five deciles = higher [32]. ^2^ Includes participants studying. ^3^ Assessed by the Accessibility Remoteness Index of Australia (ARIA) [33].

**Table 2:** Agreement between camera and accelerometer data by means (95% CI), equivalence testing, intraclass correlation coefficient and Spearman’s rho, *n* = 636 person-hours, *n* = 53 participants.

Episode	Mean (95% CI) Camera (min/h) ^1^	Mean (95% CI) Accelerometer (min/h) ^2^	Accelerometer Region of Equivalence ^3^	Equivalence Test *t*-Value ^3^	Equivalence Test *p*-Value ^3^	ICC (95% CI) ^4^	Correlation (Spearman’s)	Correlation (Spearman’s) *p*-Value
Sedentary	34 (29,39)	42 (40,43)	37,46	−22.3	1.00	0.81 (0.78,0.84)	0.77	<0.001
Light	18 (13,23)	12 (11,13)	11,13	11.8	1.00	0.55 (0.48,0.62)	0.59	<0.001
MVPA	5 (2,8)	7 (6,7)	6,7	−5.8	1.00	0.52 (0.44,0.59)	0.51	<0.001

^1^ Coded using the 2011 Compendium of Physical Activities (Compendium) [16]. Camera captures images approximately every 30-s i.e., the first two images every minute were included in the analysis. ^2^ Using Montoye (2020) cut-points [12]. Counts per minute (CPM); 2860 (sedentary), 2860–3940 (light) and ≥ 3941 (MVPA). ^3^ Equivalence testing was conducted using two one sided *t* tests. ^4^ Intraclass correlation coefficient (ICC, two way mixed; consistency); *p* < 0.05 considered statistically significant. CI indicates confidence interval.

**Table 3:** Minutes of sedentary, light and moderate-to-vigorous physical activity for the camera and accelerometer with the proportion of time spent in each activity intensity, *n* = 636 person-hours; *n* = 53 participants.

Activity Intensity	Camera ^1^		Accelerometer ^2^	
	Total Minutes ^3^	Mean Proportion of Time (%) ^4^	Total Minutes	Mean Proportion of Time (%) ^4^
Sedentary	21,339	59	26,428	69
Light	11,533	32	7603	20
MVPA	3109	9	4130	11
Total	35,981	100	38,161	100

^1^ Coded using the Compendium of Physical Activities (Compendium) [16]. Sedentary behavior defined as ≤1.5 METs, light activity between 1.6–2.9 METs and moderate-to-vigorous activity (MVPA) ≥3 METs. ^2^ Using Montoye (2020) cut-points. Counts per minute (CPM); <2860 (sedentary), 2860–3940 (light) and ≥ 3941 (MVPA) [12]. ^3^ Camera captures images approximately every 30-s i.e., the first two images every minute were included in the analysis. ^4^ Minutes of physical activity (PA) intensity divided by device wear-time.

## Supplementary Materials

The following are available online at https://www.mdpi.com/1660-4601/17/24/9323/s1, Table S1: Agreement between camera and accelerometer data by means (95% CI), equivalence testing, intraclass correlation coefficient and Spearman’s rho, n = 636 person-hours, n = 53 participants using Freedson (1998) cut-points.
