# Supplementary material for: Feasibility Study Comparing Physical Activity Classifications from Accelerometers with Wearable Camera Data
Source: Int J Environ Res Public Health. 2020 Dec 13;17(24):9323. doi: 10.3390/ijerph17249323 (PMC7764508; doi:10.3390/ijerph17249323)
Supplement: Supplementary file 1 [file ijerph-17-09323-s001.pdf]

Supplementary Table S1. Agreement between camera and accelerometer data by means (95% CI), equivalence testing, intraclass correlation coefficient and Spearman's rho, *n* = 636 person-hours, *n* = 53 participants.

Episode	Mean (95% CI) Camera (min/h)[1]	Mean (95% CI) Accelerometer (min/h)[2]	Accelerometer Region of Equivalence[3]	Equivalence Test *t*-Value[3]	Equivalence Test *p*-Value[3]	ICC (95% CI)[4]	Correlation (Spearman's)	Correlation (Spearman's) *p*-Value
Sedentary	34 (29,39)	19 (16,22)	17, 21	26.8	1.00	0.74 (0.70,0.78)	0.66	<0.001
Light	18 (13,23)	30 (28,32)	27 ,33	-21.0	1.00	0.39 (0.29,0.48)	0.27	<0.001
MVPA	5 (2,8)	11 (8,14)	10, 12	-16.7	1.00	0.64 (0.58,0.69)	0.55	<0.001

[1]Coded using the 2011 Compendium of Physical Activities (Compendium) [16]. Camera captures images approximately every 30-s i.e., the first two images every minute were included in the analysis [2]Using Freedson (1998) cut-points [11]. Counts per minute (CPM); Sedentary 0 – 99; Light 100 – 1951; Moderate-to-vigorous physical activity (MVPA) ≥1952 [3] Equivalence testing was conducted using two one sided *t* tests [4]Intraclass correlation coefficient (ICC, two way mixed; consistency); $p < 0.05$ considered statistically significant. CI indicates confidence interval.
